# Deciphering embryo-maternal cross-talk: advanced in vitro approaches

**DOI:** 10.1590/1984-3143-AR2025-0059

**Published:** 2025-08-13

**Authors:** Gislaine dos Santos, María Gemma Millán de la Blanca, Yulia Nathaly Cajas, Rosane Mazzarella, Karina Cañón-Beltrán, Maria Encina Gonzalez Martínez, Dimitrios Rizos

**Affiliations:** 1 Departamento de Reproducción Animal, Instituto Nacional de Investigación y Tecnología Agraria y Alimentaria – INIA, Consejo Superior de Investigaciones Científicas – CSIC, Madrid, Spain; 2 Departamento de Anatomía y Embriología, Facultad de Veterinaria, Universidad Complutense de Madrid – UCM, Madrid, Spain; 3 Departamento de Ciencias Biológicas, Universidad Técnica Particular de Loja – UTPL, Loja, Ecuador

**Keywords:** embryo-maternal communication, *in vitro* systems, extracellular vesicles, reproductive biology

## Abstract

Embryo-maternal communication is a critical process that influences early embryonic development, implantation success, and pregnancy outcomes across mammalian species. This review examines the diverse *in vitro* systems developed to study this complex dialogue, highlighting their applications, advantages, and limitations. We explore conventional approaches such as two-dimensional (2D) cell cultures, which despite their simplicity, face challenges in replicating the three-dimensional (3D) architecture and cellular functions present *in vivo*. The review progresses through increasingly sophisticated models, including fluid co-culture systems that incorporate bioactive molecules, explant cultures that maintain tissue architecture, air-liquid interface systems that promote epithelial polarization and differentiation, 3D organoid systems that recapitulate complex structural organization, and organ-on-a-chip platforms that recreate mechanical forces and dynamic conditions. Special attention is given to the emerging role of extracellular vesicles (EVs) as mediators of embryo-maternal communication, transporting crucial molecular signals between the embryo and reproductive tract. By comparing these systems across species and developmental stages, we provide a comprehensive framework for selecting appropriate models based on specific research questions. The integration of these *in vitro* approaches with advanced analytical techniques offers promising avenues for understanding embryo-maternal cross-talk, potentially leading to improved assisted reproductive technologies and strategies to mitigate early pregnancy loss. As technology advances, the continued refinement of these systems will further illuminate the intricate molecular and cellular mechanisms underlying successful embryo development and implantation.

## Introduction

**Figure 1:**
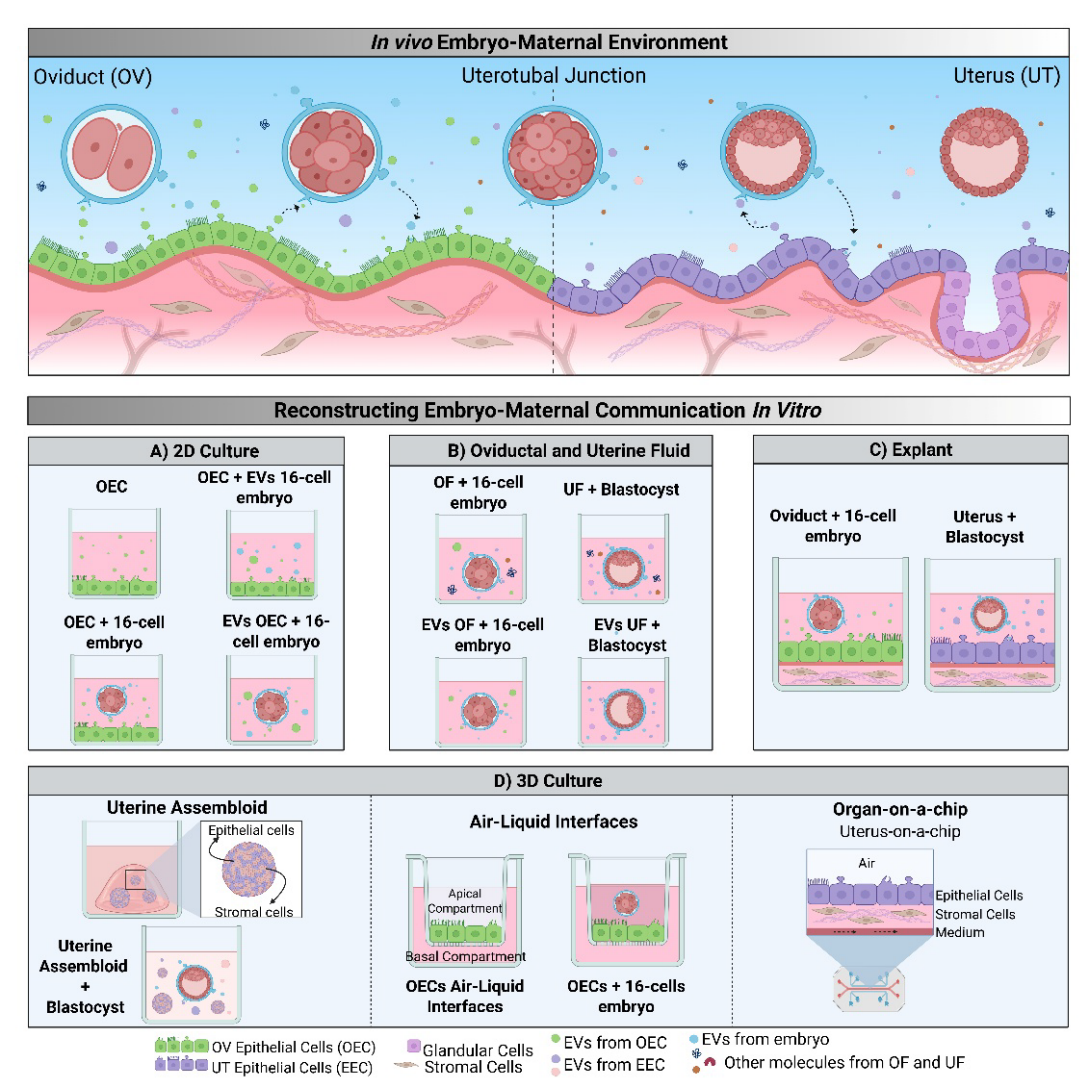
Schematic representation of in vivo embryonic development, from the oviduct to the uterus, and different in vitro approaches to mimic early embryo-maternal communication. In vivo, the zygote develops in the oviduct, interacting with OECs and EVs. After reaching the uterus via the uterotubal junction, it communicates with the endometrium to initiate implantation. In both environments, bidirectional communication is mediated by EVs and other molecules present in the luminal fluid. Several in vitro models aim to mimic these interactions: (A) 2D cultures with co-culture of OECs and embryos (16-cell or blastocyst stage), or with EVs derived from OECs, endometrial epithelial cells (EECs), or embryos; (B) Embryo culture in OF, UF, or with EVs isolated from these fluids; (C) Oviduct or uterine explants co-cultured with embryos at appropriate developmental stages; (D) Advanced 3D systems, including uterine assembloids (epithelial + stromal cells), OECs grown at an air-liquid interface to mimic the oviductal epithelium, and organ-on-a-chip platforms (e.g., uterus-on-a-chip) that recreate physiological uterine microenvironments.

Pregnancy loss affects all mammalian species, particularly during early embryo development. In cattle, early gestational losses are a significant challenge, with approximately 30% of pregnancies failing before day 7. In beef cattle, about 28% of losses occur by day 7, followed by an additional 15.6% later (Reese et al., 2020). In high-producing dairy cows, 20-50% of pregnancies are lost within the first week, with further losses (~30%) reported between days 8 and 27 (Wiltbank et al., 2016). In seasonally bred grazing dairy cows, ~29% of pregnancies fail by day 7, with a cumulative loss of 40.9% by day 15 and minimal losses beyond that point (Berg et al., 2022). In humans, up to 60% are lost in the early stages (Reese et al., 2020; Ealy and Seekford, 2019; Larsen et al., 2013). A critical factor contributing to these losses is the disruption in embryo-maternal communication (Rizos et al., 2002). Although embryos can develop *in vitro*, disruptions to this early dialogue can lead to poor pregnancy outcomes, including low blastocyst formation rates (30-40%), altered gene expression patterns, imbalances in the ratio between the inner cell mass (ICM) and the trophectoderm (TE), and lower pregnancy rates (Pontes et al., 2009; Lonergan et al., 2003; Rizos et al., 2002). Therefore, understanding the mechanisms of embryo-maternal communication is essential for identifying factors that influence embryonic development. However, ethical and practical considerations have led to a growing emphasis on reducing the use of animals and humans in research (Kiani et al., 2022). Much of our knowledge about early embryo-maternal communication stems from *in vivo* studies, which underscores the need for developing technologies that can effectively mimic these interactions. This review aims to contextualize the current knowledge regarding embryo interactions within the oviduct and uterine environments, identify gaps in technical approaches, and discuss emerging technologies (Figure 1) that may enhance our understanding and mitigate losses during early embryonic development.

## Setting the stage: early embryo-maternal communication *in vivo*

In cattle and other mammals, the first stages of embryo development occurs in the oviduct (Figure 1). During these initial days of development, the fertilized zygote undergoes a series of specialized divisions and metabolic changes that culminate in embryo genome activation (EGA), which typically begins at the 2-cell stage in mice, 4-cell to 8-cell in humans and 4-cell to 16-cell stages in bovine (Lonergan and Fair, 2014; Graf et al., 2014; Vassena et al., 2011; Taylor et al., 1997; Flach et al., 1982). Over the course of three and a half to four days in the oviduct, the embryo is supported by ciliated and secretory cells, as well as oviductal fluid (OF). These components play key roles by secreting substances, growth factors, and extracellular vesicles (EVs), while also creating a directed flow that facilitates embryo transport to the uterus via the utero-tubal junction (as revised by Bastos et al., 2022; Ghersevich et al., 2015). Once the embryo reaches the uterus at the morula stage, it continues to develop through the blastocyst, which consists of a fluid-filled cavity called the blastocele, a smaller compact inner cell mass (ICM: which forms the fetus), and an outer epithelial layer, the trophectoderm (TE: which forms the extraembryonic membranes). By day 9 in cattle, the blastocyst will hatch out of the zona pellucida, interact with the endometrium, and implantation begins around days 16-20 (Spencer, 2013). This differs from humans, where the implantation begins immediately after hatching during the processes of apposition (Kim and Kim, 2017). Throughout this period, the uterine environment-comprising endometrial cells and uterine fluid (UF) provides nutrients and molecular signals, including EVs, that are essential not only for embryonic growth but also for implantation and maternal recognition of pregnancy.

## 2D culture systems for studying early embryo-maternal cross-talk

The most commonly reported method for investigating embryo-maternal communication *in vitro* is the association of *in vitro* embryo production (IVEP) with 2D monolayer culture (Figure 1A). Methods to culture the oviduct epithelial cells (OECs) and endometrial epithelial cells (EECs) can differ concerning cell isolation techniques, culture conditions, duration, the culture medium used, and supplements included (Cronin et al., 2012; Ulbrich et al., 2010). Although the 2D monolayer culture offers a straightforward and low-maintenance method requiring minimal resources, it is critical to note that it quickly loses normal cellular function due to differentiation or senescence, thereby reducing its biological similarity to *in vivo* cells (Table 1A). Nevertheless, it can still provide valuable insights into embryo-maternal interactions during preimplantation embryonic development as discussed above.

**Table 1:** Advantages, Disadvantages, and Typical Applications of In Vitro Systems for Studying Embryo-Maternal Communication.

**In vitro systems**	**Advantages**	**Disadvantages**	**Typical applications**
**A) 2D**	- Low costs	- Cells tend to dedifferentiate	- Basic cell function
	- Quick to grow	- Lack of a complex extracellular matrix	- Initial screening of embryo and maternal cells factors
	- Easy to use	- Poor recapitulation of *in vivo* gradients	
	- Easy downstream processing		
**B) Total Fluids**	- Better mimics the dynamic in vivo environment via fluid	- High variability in fluid composition	- Effects of reproductive tract secretions on embryo development
	- Incorporates natural bioactive molecules	- Batch-to-batch inconsistency	
**C) EVs**	- Concentrate essential bioactive factors	- Heterogeneity in EV population	- Bidirectional embryo-maternal communication
	-Reduced unwanted variability	- Limited understanding of uptake mechanisms	- Role of EVs cargo
	- Mediate targeted cell-to-cell communication		- Embryo IVC supplement
**D) Explant**	- Retain native tissue architecture and cell diversity	- Limited viability	- Short-term studies of tissue-level responses to embryo presence
	- Reflect in vivo organization	- Short window for experimentation	- Investigating local tissue remodeling during implantation
	- Preserves cell-cell and cell-matrix interactions	- Difficult to control culture conditions consistently	- Examining the interaction between embryos and intact tissue segments
	- Maintains tissue-specific functions		
**E) Air-liquid interface**	- Allows for apical secretion of factors	- Technical complexity in setup and maintenance	- Modeling the interface between luminal fluid and epithelial cells
	- Supports development of functional epithelial barriers	- Requires specialized culture inserts	- Long-term culture of differentiated reproductive tract epithelia
**F) 3D**	- Complex structural organization	- Limited access to the luminal compartment	- Recreating the complex architecture of reproductive tissues
	- Maintain cell polarity and differentiation	- Variability in size and formation	- Studying long-term embryo-maternal interactions
	- Support long-term culture	- More complex to establish than 2D cultures	
	- Better recapitulates in vivo tissue architecture	- Higher technical expertise required	
		- More resource-intensive	
**G) Organ-on-a-chip**	- Recapitulate the in vivo interactions	- Complex design and fabrication process	- Dynamic aspects of embryo-maternal communication
	- Ability to recreate mechanical forces	- Requiring specialized equipment and expertise	- Recreating the fluid dynamics of the reproductive tract
	- Easy for co-culture	- Higher costs compared to simpler models	- Integrating multiple tissue compartments in a single system
	- Precise control of biochemical and physical cues	- Technical challenges in integration	
	- Supports long-term culture	- Limited commercial availability	

### Oviductal epithelial cells in 2D culture: a model for embryo co-culture

OEC cultures have shown that early developing embryos not only use the oviductal environment to travel to the uterus but also actively shape early embryo-maternal communication (Hamdi et al., 2019; Schmaltz-Panneau et al., 2015; Cordova et al., 2014; Edwards et al., 1997). For example, OECs support early embryonic development by secreting embryotrophic growth factors, removing embryotoxic substances, and preventing the formation of reactive oxygen species (ROS) (Edwards et al., 1997; Nancarrow and Hill, 1994; Thompson et al., 1990). Moreover, co-culturing embryos with OECs prevents the blocking of EGA, and increases both the number and quality of blastocysts (Schmaltz-Panneau et al., 2015; Cordova et al., 2014; Ellington et al., 1990; Gandolfi and Moor, 1987). Additionally, it provides evidence that early embryos modify the OECs transcriptome with the cellular response varying according to the embryos developmental stage, likely due to OECs adapting their transcriptomic profile in response to embryonic signaling (Hamdi et al., 2019; Schmaltz-Panneau et al., 2014). Furthermore, embryo-oviduct interactions induce specific changes in the transcriptional levels of bone morphogenetic protein (BMP) signaling (García et al., 2017), resulting in a bidirectional response that offers insights into early maternal-embryonic communication. Additionally, embryos stimulate an anti-inflammatory response in the OECs that in turn prompts the 16 cell-embryos to secrete interferon-tau (IFNT), thereby modulating gene expression in peripheral blood mononuclear cells (PBMCs) (Talukder et al., 2018). Altogether, these data suggest that culturing of the OECs as a monolayer has the potential to provide useful information on embryo-oviduct interactions during early embryonic development.

### Endometrial epithelial cells in 2D culture: a model for embryo co-culture

In addition to the role of the oviduct, the 2D system helped to understand that the embryo actively shapes the local uterine environment. Studies using 2D cultures of EECs have revealed that, as early as day 7 of pregnancy, the embryo can locally modify endometrial function (Sponchiado et al., 2020; Allen and Wright, 1984). Notably, this is not only mediated through the secretion of bioactive molecules but also through physical contact between the embryo and EECs, thereby intensifying their reciprocal molecular interactions (Sponchiado et al., 2020). Further complexity in this embryonic dialogue is highlighted by studies indicating that both embryo quality and embryo sex influence the transcriptional profile of EECs. For example, high-quality embryos have been shown to modify the secretion pattern of IL-8 in EECs, a cytokine vital for preparing the uterus for implantation, while EECs exhibit differential gene expression (e.g., CTGF, SLC2A1, and SLC2A5) in response to embryonic signals (Gómez et al., 2018; Caballero-Campo et al., 2002). Additionally, the conditioned media from both OECs and EECs enhance the developmental kinetics of *in vitro*-produced embryos (Devi et al., 2022; Sidrat et al., 2020), suggesting that both direct cell contact and secreted factors play crucial roles for early embryonic development.

### Extracellular vesicles from 2D oviductal and uterine cells: insights from co-culture models

Recent studies indicate that EVs released by OECs and EECs play a key role in mediating the beneficial effects observed in the co-culture of embryos with 2D cells (Figure 1A). Conditioned medium from OECs and their EVs during IVC has been shown to improve blastocyst yield and hatching capacity, promote embryo development at the 8-16 cells stage, upregulate genes related to development and implantation, and enhance both cellular proliferation and cryotolerance (Sidrat et al., 2020; Lopera-Vásquez et al., 2016). Furthermore, embryo quality appears to influence the oviductal environment reciprocally. EVs derived from high-quality embryos induce specific transcriptional changes in OECs, such as the upregulation of genes like ISG-15, MX1, and OAS1Y (Dissanayake et al., 2021). This observation supports a model in which OECs function as sensors of embryonic quality, potentially modulating the miRNA content of their secreted in response to embryonic signals (Hamdi et al., 2024). Collectively, these findings suggest that EVs facilitate a dynamic bidirectional communication between embryos and OECs, enabling the OECs to finetune their secretory profile according to embryonic competence.

The uterine compartment plays a critical role in this crosstalk, with EVs derived from EECs shown to enhance embryonic development by increasing embryo diameter and promoting blastocyst expansion and hatching in bovine models (Aguilera et al., 2024). In addition, preimplantation embryos release EVs that interact with EECs, altering their transcriptional profile, most notably, increasing OXTR expression in response to EVs released by *in vitro*-produced embryos during days 7 to 9 (Aguilera et al., 2023). Together, these studies suggest that embryos actively signal to the EECs via EVs, while the EECs also communicate with the embryo, modulating gene expression in a way that may influence maternal recognition of pregnancy.

## Oviductal and uterine fluids for studying early embryo-maternal cross-talk

Considering the importance of this microenvironment *in vivo*, supplementing culture media during IVC with reproductive fluids, such as OF and UF, has been explored as a strategy to better replicate the *in vivo* environment, enhancing our understanding of embryo-maternal communication (Figure 1B). The use of OF during IVC has been shown to increase the blastocyst rate, cryotolerance, and the number of trophoblast cells, likely due to the co-culture induced changes in the abundance of transcripts related to glucose and lipid metabolism, epigenetic regulation, and water channel expression (Lopera-Vásquez et al., 2017b; Archibong et al., 1989). Furthermore, a sequential supplementation approach—using OF during the early days (1-4) and UF during the later days (4-9) of IVC—has been demonstrated to support embryo development and improve embryo quality (Hamdi et al., 2018). Additionally, it has been shown that UF may exhibit antioxidant activity, reducing the accumulation of ROS in blastocysts (Hamdi et al., 2018). While reproductive fluids can represent the *in vivo* environment, their complex and variable molecular composition may result in inconsistent effects on embryonic development (Table 1B). In contrast, isolating EVs from OF and UF provides a more defined and reproducible approach (Table 1C). These EVs are enriched in essential bioactive factors critical for targeted cell-to-cell communication and signaling, thereby reducing unwanted variability. This strategy enables more precise modulation of the culture environment, ultimately promoting more consistent and favorable outcomes in embryonic development.

### Extracellular vesicles from oviductal and uterine fluid: modulating early embryo development and maternal recognition

Recent studies have demonstrated that supplementing IVC with EVs from both OF and UF fluids (Figure 1B) can improve key developmental parameters, such as blastocyst formation rates, blastocyst diameter, and total cell numbers, while also reducing apoptosis and stress within the embryo. For instance, a higher total cell number, which correlates with improved viability and implantation potential, was observed in blastocysts treated with OF and UF-EVs, regardless of whether BSA or dFCS was used as a protein supplement (Li et al., 2023; Leal et al., 2022). Similarly, UF-EVs significantly reduce apoptotic cell numbers in SCNT-derived blastocysts and improve the ratio of ICM to TE, highlighting the protective and supportive role of these EVs (Qiao et al., 2018). Beyond these quantitative improvements, EVs also modulate the molecular environment of the embryo. Early exposure to OF-EVs can alter the embryonic transcriptome, enriching pathways related to metabolism, cell communication, and apoptosis (Alcântara-Neto et al., 2022). Additionally, EVs treatment during IVC modifies the lipid profile of embryos, increasing key phospholipids such as PC (phosphatidylcholine), PE (phosphatidylethanolamine), and SM (sphingomyelin), characteristic of *in vivo*-produced embryos (Leal et al., 2022; Banliat et al., 2020).

Uterine EVs appear to act at a later stage of embryo development. Aguilera et al. (2024) found that UF-EVs upregulate IFNT expression and increase embryonic diameter, suggesting an critical role in embryo-maternal signaling and recognition of pregnancy. Furthermore, studies from our group (Cañón-Beltrán et al., 2024; Mazzarella et al., 2024) suggest that miRNAs within EVs fine-tune gene expression related to lipid metabolism and stress responses, thereby further supporting embryo viability and enhancing cryotolerance, as evidenced by improved post-vitrification survival rates (Leal et al., 2022; Lopera-Vásquez et al., 2017a). In summary, the complementary actions of EVs from the oviduct and uterus create a more favorable environment for embryo development. OF-EVs primarily modulate the embryonic transcriptome and lipid composition during early stages (from 2 cells to morula), whereas UF-EVs play a pivotal role during blastocyst expansion by enhancing growth, reducing apoptosis, and promoting effective embryo-maternal communication.

## Explant culture system for studying early embryo-maternal cross-talk

Explants provide an alternative to fluid-based models and 2D culture systems by preserving native tissue architecture, including cell-cell interactions and the extracellular matrix, thus offering a more physiologically relevant environment that more accurately mimics the *in vivo* embryo-maternal dialogue (Table 1D). For instance, co-culturing endometrial explants with Day-8 blastocysts (Figure 1C) for 6 hours leads to a regulation of interferon-stimulated genes (ISGs) (Passaro et al., 2019, 2018). Interestingly, even without direct contact, conditioned medium from blastocyst cultures was sufficient to elevate ISG expression, with the response intensifying as the number of blastocysts increased (Passaro et al., 2018). These findings highlight the pivotal role of IFNT, secreted by the embryo, as one of the earliest signals alerting the maternal system to the presence of pregnancy. Additionally, a brief exposure to blastocysts, regardless of being produced *in vivo* or *in vitro* (Bridi et al., 2025), alters the global transcriptomic profile of oviductal (Mazzarella et al., 2025) and endometrial explants (Bridi et al., 2025; Passaro et al., 2019), noted through the release of secreted molecules (Passaro et al., 2019) including EVs with distinct miRNA profile (Bridi et al., 2025) and proteins (Mazzarella et al., 2025). This underscores the potency of embryonic signals in orchestrating maternal responses, even without direct tissue contact.

One of the main limitations of explant models, especially in bovine studies, is their limited viability (Table 1D). Additionally, controlling culture conditions is more challenging in explant systems compared to 2D or 3D models. For example, although explants cannot be maintained for extended periods, limiting their use in long-term studies of embryo-maternal interactions, a 6-hour culture period has been shown to be sufficient to elicit responses to embryonic presence while preserving the structural and functional integrity of the tissue (Passaro et al., 2018). However, by 48 hours of culture, significant necrotic fragmentation of glands and stroma is observed in the center of the explants (Borges et al., 2012). These limitations have driven the need of development of advanced 3D culture models, such as organoid technologies and organ-on-a-chip platforms, which better preserve cellular differentiation over extended periods, support fluid flow and mechanical forces, and provide a more stable and physiological environment for studying embryo-maternal interactions (Table 1F).

## Air-liquid interfaces

By culturing epithelial cells on porous membranes at an air-liquid interface (Figure 1D), these models promote cell differentiation and establish apical-basolateral polarity, generating a luminal microenvironment enriched in secretions that closely mirrors *in vivo* OF (Chen and Schoen, 2019). This compartmentalization allows researchers to distinguish signals available to gametes and embryos (luminal secretions) from those interacting with sub‐epithelial tissues (basolateral secretions), and to monitor dynamic fluid changes regulated by progesterone and estradiol (Chen et al., 2013, 2018). Such hormone‐driven modulation of the culture milieu offers insights into how the early embryonic environment may influence developmental competence and epigenetic programming (El Hajj and Haaf, 2013; Lucas, 2013). Building on these advances, sequential co‐culture approaches—first with OECs, then with EECs—have demonstrated improvements in both blastocyst yield and quality (van der Weijden et al., 2017). Remarkably, bovine embryos cultured solely within air-liquid interface OEC systems can reach the blastocyst stage without classical culture media, exhibiting gene‐expression profiles linked to methylation, differentiation, and apoptosis (van der Weijden et al., 2017). Proteomic analysis of air-liquid interface‐derived fluid has uncovered over 3,000 proteins shared with OF, underscoring the physiological relevance of these models (Chen et al., 2017). Looking forward, integrating air-liquid interface cultures with microfluidic “oviduct‐on‐a‐chip” technologies promises to refine fluid dynamics and hormone delivery further—paving the way for more predictive models of fertilization, cleavage, and implantation in assisted reproductive technologies (Ferraz et al., 2017).

## Oviductal and endometrial 3D culture models: organoids, spheroids and assembloids in early embryo-maternal communication

Three dimensional culture systems address key limitations of both 2D models and explant cultures (Table 1). While 2D models often fail to replicate the complex spatial organization, cell-cell interactions, and physiological gradients found *in vivo*, explant cultures maintain architecture but are limited by short-term viability. In contrast, 3D culture systems closely mimic the *in vivo* environment by promoting cell-cell and cell-matrix interactions as well as establishing biochemical and mechanical gradients. Therefore, different 3D models have been developed to address various research needs. Spheroids are spherical cell aggregates that are cultured in suspension for a few days mainly for the study of cell behavior (Pranomphon et al., 2024b). In contrast, organoids offer a higher tissue specificity, exemplified by oviductal organoids composed predominantly of epithelial cells, and endometrial organoids which also include glandular cells (Kwon et al., 2025). Taking complexity a step further, assembloids (Figure 1D) incorporate multiple cell types (including epithelial, glandular, and stromal cells) to more accurately recapitulate the native tissue’s structure and function (Crawford et al., 2024; Rawlings et al., 2021).

Both organoids and assembloids models are commonly cultivated using acellular scaffolds made from biological or synthetic origins. Scaffold-based systems include: 1) collagen scaffolds, that mimic the natural extracellular matrix (Abbas et al., 2020), 2) matrigel, cultrex and geltrex, a biological hydrogel that provides structural support and promotes cell signaling (Ahmad et al., 2024; Lawson et al., 2023; Ford et al., 2021; Kessler et al., 2015) and 3) synthetic scaffolds such as polyethylene glycol (PEG), which offer tailored mechanical properties (Gnecco et al., 2023). On the other hand, scaffold-free models, such as: 1) spheroids generated by hanging drop methods, 2) low-attachment plates or gels, 3) agitation-based formation, and 4) magnetic levitation, are also widely used (Langhans, 2018).

Although 3D culture can preserve the morphology of the oviduct and endometrium, as well as the polarization of OEC and EEC, it still has limitations (Table 1F). The luminal compartment of the organoid, spheroids, or assembloids is only accessible for gametes or embryos via micro-puncturing. This barrier may hinder the direct interaction between embryos and the apical surface of the cells, which is crucial for embryo-maternal communication. To address this, strategies such as apical-out reversal protocol (Co et al., 2021), vigorous pipetting (van der Sanden et al., 2018) and suspension culture (Ahmad et al., 2024) have been developed to expose the apical surface. Another consideration is that the intra-organoid fluid has been shown to have a distinct biochemical composition compared to the extra-organoid fluid and culture medium (Simintiras et al., 2021). This difference can affect embryo-epithelium communication by altering the availability of essential signals like growth factors, cytokines, and EVs. Thus, while 3D secretions can offer an advantage by recreating a more physiological microenvironment, they may also introduce variability if not carefully characterized and controlled. Nevertheless, these models still provide valuable insights into embryo-maternal interactions during preimplantation embryonic development, as discussed above.

### Oviductal epithelial organoids and spheroids

Oviductal epithelial organoids and spheroids have been employed to explore the cellular and molecular mechanisms within the non-pregnant oviduct, as well as during early pregnancy stages (Day 1 to 5 post-fertilization in bovine). Recent studies have established these models across various species, including humans (Kessler et al., 2015), mice (Xie et al., 2018), porcine (Lawson et al., 2023), canine (Lawson et al., 2023), feline (Lawson et al., 2023; Thompson et al., 2023), equine (Lawson et al., 2023; Thompson et al., 2022), turkey (Santativongchai et al., 2024) and bovine (Pranomphon et al., 2024a, b; Lawson et al., 2023). These models highlight the versatility of oviductal spheroid and organoid systems, supporting comparative studies and enhancing our understanding of reproductive physiology across species.

Spheroid models have been explored as a supportive IVC system for embryo production, highlighting their potential to enhance embryo-maternal communication and embryo quality. For instance, the co-culture of embryos with spheroids improved embryo development and blastocyst quality, particularly under high oxygen conditions, by mitigating oxidative stress (Pranomphon et al., 2024a). The presence of embryos also induced morphological changes in the spheroids, such as an increased proportion of vesicle-shaped structures (Pranomphon et al., 2024a). Moreover, while some genes (ANXA1, ESR1, HSPA8, and HSPA1A) remained stable, others (OVGP1 and PGR) decreased over time, suggesting that further refinements, like the supplementation of reproductive steroid hormones, might improve the physiological relevance of the model (Pranomphon et al., 2024a). Furthermore, this study indicate that spheroid models not only support embryo development but also adapt to embryonic signals, thus simulating the dynamic *in vivo* environment and highlighting areas for further model refinement.

Additionally, several studies have shown that oviduct organoids replicate the native architecture by maintaining different types of epithelial cells, including ciliated and secretory cells (Lawson et al., 2023; Kessler et al., 2015). Moreover, these models also contain progenitor cells that support long-term culture, which is essential for studies of oviduct function and response to environmental challenges. In this context, Menjivar et al. (2023) investigated the effects of heat shock on oviduct organoids, revealing significant molecular changes. They found that heat shock activated key genes such as COX1, ACTB, CST6, TPT1, and HSPB1, and modified the EVs cargo, altering miRNAs such as miR-1246, miR-148a, miR21-5p, miR-451, and miR-92a. These results suggest that heat shock may disrupt the oviductal microenvironment by modifying both gene expression and EV content, potentially impacting fertilization and early embryo development (Menjivar et al., 2023). Together, these findings highlight the potential of oviduct organoids as a powerful model to unravel molecular responses to environmental challenges.

### Endometrial organoids: epithelial and glandular models 

Similarly, endometrial epithelial organoids have been widely used to study uterine biology and embryo-maternal communication. Recent studies have established organoid models derived from various species, including humans (Turco et al., 2017), mice (Kwon et al., 2025; Boretto et al., 2017), canines, felines, porcines, and bovines (Kwon et al., 2025). The endometrial epithelial organoid models contained a polarized columnar epithelium surrounding a lumen and displayed similar molecules, and functional properties that closely recapitulate the endometrial tissue (Luddi et al., 2020). Their long-term culture capability and responsiveness to hormonal stimulation allow them to mirror the dynamic changes of the luminal epithelium during the menstrual cycle (Luddi et al., 2020; Fitzgerald et al., 2019).

Glandular organoids further contribute to our understanding by providing a complementary perspective on endometrial structure and function. Their ability to acquire a decidual-like phenotype in response to pregnancy signals highlights their potential as a robust experimental platform for studying early embryo-maternal communication. In particular, by exposing glandular organoids to pregnancy signals such as cyclic AMP, prolactin (from the stroma), and hCG and hPL (from the trophoblast), they begin to exhibit phenotypic characteristics of decidual tissue similar to those of early pregnancy (Turco et al., 2017).

Importantly, these organoids have shown that the embryo can interact with the endometrial environment at the molec ular level. For example, organoids treated with a conditioned medium from successfully implanted embryos expressed a new, more acidic glycoform of GdA (Luddi et al., 2021), suggesting that the embryonic secretome may influence adhesion and signaling molecules critical for implantation. Together, these findings highlight the relevance of endometrial epithelial and glandular organoids as dynamic, physiologically relevant models for investigating how the maternal endometrium responds to embryonic signals. Their ability to replicate hormonally driven transformations and interact with embryonic cues *in vitro* makes them powerful tools for advancing our understanding of early pregnancy events and implantation.

### Advances with assembloids

With the recent development of oviductal and endometrial organoids and spheroids, assembloids emerge as more complex 3D models that combine different cell types. In the case of embryo-maternal communication, oviductal assembloids can integrate epithelial and stromal components, while uterine assembloids can integrate epithelial, glandular, and stromal components, along with trophoblast cells (from the embryo). For instance, by combining oviductal epithelial cells and stromal cells, the assembloids exhibited a significantly higher number of extracellular matrix proteins compared to standard organoids, as well as a physiological response to steroid hormone stimulation (β-estradiol and progesterone) (Crawford et al., 2024). This response was evidenced by changes in the expression of hormonal receptors (ESR1, PGR) and cellular differentiation markers (PAX8, FOXJ1, OLFM4) (Crawford et al., 2024). Moreover, functional cilia were observed at the epithelium-lumen interface, with their activity enhanced by β-estradiol stimulation (Crawford et al., 2024), thereby capturing the organ's mechanical function.

Additionally, by incorporating both glandular epithelium (in the form of endometrial organoids) and primary stromal cells into an assembloid system and co-culturing with human embryos, crucial insights into embryo-maternal communication were gained. Specifically, the presence of senescent decidual cells significantly influences the movement of stromal cells toward the expanding embryo, creating a permissive environment for embryo attachment (Rawlings et al., 2021). In an embryo that has attached in this environment, the expression of OCT4 and GATA6 markers was observed in the epiblasts and hypoblasts, which is indicative of early embryonic development following implantation and is considered essential for pregnancy (Rawlings et al., 2021). Additionally, the analysis of secreted factors during co-culture, such as human chorionic gonadotropin (hCG) released by the embryo and various cytokines and chemokines produced by the assembloid cells (Rawlings et al., 2021), enabled the investigation of the intricate molecular communication that occurs between the embryo and the endometrium during the early stages of implantation. Altogether, these findings demonstrate that assembloids provide a robust and physiologically relevant platform to study embryo-maternal communication. By incorporating multiple interacting cell types, these models more closely mimic the complex cellular, mechanical, and molecular environment of the reproductive tract.

## Organs-on-a-chip

### Oviduct-on-a-chip

The microfluidic device that mimics the bovine oviduct epithelium was described in different species, including bovine (Ferraz et al., 2018, 2017), humans (Zha et al., 2023; Jackson-Bey et al., 2020; Xiao et al., 2017), canines (Ferraz et al., 2020) and mice (Wang et al., 2022). One significant advance in this field is the observation that the presence of fluid flow may sustain ciliary activity in BOECs for 2 to 6 weeks (Jackson-Bey et al., 2020; Ferraz et al., 2017). This represents an improvement over traditional 2D cultures and explants, which often lose ciliary function and have limited viability, respectively. Additionally, this system can replicate the hormonal dynamics of the estrous cycle, including follicular and luteal phases (Ferraz et al., 2018). The oviduct-on-a-chip has been applied to both *in vitro* fertilization (IVF) and embryo culture. In bovine IVF, microfluidic devices have shown a reduced polyspermy rates compared to 2D culture systems and conditions without oviductal cells (Ferraz et al., 2017), suggesting improved control over fertilization conditions. However, when used for embryo culture in the same species, fluid flow led to zygote displacement, impairing early embryonic development (Ferraz et al., 2018). In mice, embryo culture within the device showed no significant differences in developmental rates compared to traditional culture methods (Wang et al., 2022). However, mice embryos cultured in the device exhibited lower levels of ROS, which may indicate a less stressful and more supportive environment (Wang et al., 2022).

### Endometrium-on-a-chip

The endometrium-on-a-chip also has been reported in humans (Ahn et al., 2021; Gnecco et al., 2019, 2017) and bovine (Tinning et al., 2025; De Bem et al., 2021). The development of this system that may compartmentalize key endometrial cell types (vascular, stromal and epithelial cells) has opened new possibilities for studying embryo-endometrial communication under controlled *in vitro* conditions (Figure 1D). In particular, a microfluidic model incorporating perivascular stromal and endothelial cells, successfully recapitulated the process of decidualization, which is fundamental for endometrial receptivity. Importantly, this model supported hormonal treatment and long-term co-culture for up to 28 days, aligning with the length of a typical menstrual cycle (Gnecco et al., 2017, 2019). When physiological shear stress was applied through controlled fluid flow, endothelial cells exhibited enhanced stromal decidualization, highlighting the role of mechanical cues in modulating endometrial function (Gnecco et al., 2019). In addition to mechanical stimuli, metabolic conditions can also influence the endometrial environment. Using a similar platform, researchers demonstrated that variations in glucose and insulin levels led to changes in both gene expression and secreted proteins, suggesting that metabolic health directly impacts endometrial receptivity (De Bem et al., 2021). These findings are particularly relevant in the context of disorders such as diabetes and polycystic ovary syndrome, where altered metabolic states are common. More advanced models have incorporated 3D architecture to better mimic the structural complexity of the endometrium. One such system included three distinct layers (epithelium, stroma, and a vascular compartment) and hydrogel matrix allowing for more physiologically relevant interactions between cell types. This 3D endometrium-on-a-chip was used as a proof-of-concept model for embryo implantation, offering insights into the molecular and cellular dynamics that modulate early pregnancy (Ahn et al., 2021).

## Conclusion

Understanding early embryo-maternal communication is essential for improving reproductive technologies and deepening our knowledge of early pregnancy. This review highlights the evolution of in vitro models, from traditional 2D co-cultures to complex 3D systems and microfluidic organ-on-a-chip platforms, each contributing unique insights into the molecular and cellular dialogue between embryos and maternal tissues. While 2D models offer simplicity and accessibility, their biological limitations necessitate the development of more physiologically relevant systems. Fluid-based and explant models provide valuable information but are constrained by variability and short-term viability. In contrast, 3D culture systems, including organoids, spheroids, assembloids, and air-liquid interface models, offer enhanced structural and functional mimicry of in vivo conditions, enabling long-term studies and improved modeling of embryo-maternal interactions. Finally, organ-on-a-chip technologies represent the cutting edge of in vitro reproductive research, integrating dynamic mechanical and biochemical cues to recreate the native environment with unprecedented precision. Thus, these advanced models not only deepen our understanding of early embryonic development and maternal recognition of pregnancy, but also represent powerful tools for refining *in vitro* embryo production systems and improving fertility outcomes in both clinical and agricultural contexts.

## Data Availability

No research data was used.
